# Complete Genome Sequence of the Aerobic CO-Oxidizing Thermophile *Thermomicrobium roseum*

**DOI:** 10.1371/journal.pone.0004207

**Published:** 2009-01-16

**Authors:** Dongying Wu, Jason Raymond, Martin Wu, Sourav Chatterji, Qinghu Ren, Joel E. Graham, Donald A. Bryant, Frank Robb, Albert Colman, Luke J. Tallon, Jonathan H. Badger, Ramana Madupu, Naomi L. Ward, Jonathan A. Eisen

**Affiliations:** 1 University of California Davis Genome Center, Davis, California, United States of America; 2 Section of Evolution and Ecology, University of California Davis, Davis, California, United States of America; 3 Department of Medical Microbiology and Immunology, University of California Davis, Davis, California, United States of America; 4 Microbial Systems Division, Biosciences Directorate, Lawrence Livermore National Laboratory, Livermore, California, United States of America; 5 Department of Biochemistry and Molecular Biology, The Pennsylvania State University, University Park, Pennsylvania, United States of America; 6 University of Maryland Biotechnology Institute, Baltimore, Maryland, United States of America; 7 J. Craig Venter Institute, Rockville, Maryland, United States of America; University of Hyderabad, India

## Abstract

In order to enrich the phylogenetic diversity represented in the available sequenced bacterial genomes and as part of an “Assembling the Tree of Life” project, we determined the genome sequence of *Thermomicrobium roseum* DSM 5159. *T. roseum* DSM 5159 is a red-pigmented, rod-shaped, Gram-negative extreme thermophile isolated from a hot spring that possesses both an atypical cell wall composition and an unusual cell membrane that is composed entirely of long-chain 1,2-diols. Its genome is composed of two circular DNA elements, one of 2,006,217 bp (referred to as the chromosome) and one of 919,596 bp (referred to as the megaplasmid). Strikingly, though few standard housekeeping genes are found on the megaplasmid, it does encode a complete system for chemotaxis including both chemosensory components and an entire flagellar apparatus. This is the first known example of a complete flagellar system being encoded on a plasmid and suggests a straightforward means for lateral transfer of flagellum-based motility. Phylogenomic analyses support the recent rRNA-based analyses that led to *T. roseum* being removed from the phylum *Thermomicrobia* and assigned to the phylum *Chloroflexi*. Because *T. roseum* is a deep-branching member of this phylum, analysis of its genome provides insights into the evolution of the *Chloroflexi*. In addition, even though this species is not photosynthetic, analysis of the genome provides some insight into the origins of photosynthesis in the *Chloroflexi*. Metabolic pathway reconstructions and experimental studies revealed new aspects of the biology of this species. For example, we present evidence that *T. roseum* oxidizes CO aerobically, making it the first thermophile known to do so. In addition, we propose that glycosylation of its carotenoids plays a crucial role in the adaptation of the cell membrane to this bacterium's thermophilic lifestyle. Analyses of published metagenomic sequences from two hot springs similar to the one from which this strain was isolated, show that close relatives of *T. roseum* DSM 5159 are present but have some key differences from the strain sequenced.

## Introduction

Since the publication of the first complete genome sequence of a free-living organism in 1995 [1], genome sequence databases have grown at a staggering rate. However, despite the abundance of complete genome data currently available, a lack of phylogenetic diversity is evident. Some phyla have been heavily sampled, others are only sparsely represented, and many have been completely ignored [2], [3]. The current phylogenetic gaps in the genome databases prevent robust reconstruction of the tree of life, a crucial endeavor for our understanding of diverse ecosystems and biological mechanisms. To start to close these gaps, in 2002, we were funded as part of the National Science Foundation's “Assembling the Tree of Life” program (aTOL) to sequence the genomes of representatives of the eight phyla of bacteria that at the time had cultured representatives but no available genome sequence. Our intent was for these new genome sequences to not only further phylogenetic studies of bacteria, but to also encourage other investigators to focus on these neglected phyla.

One of the organisms we selected for sequencing was *Thermomicrobium roseum*, an extremely thermophilic bacterium originally isolated from Toadstool Spring, an alkaline siliceous hot spring in Yellowstone National Park [4]. This rod-shaped, non-motile, Gram-negative bacterium grows optimally at 70–75°C and pH 8.2–8.5, and is currently classified as an obligately aerobic heterotroph. In contrast to other known bacteria, its cell membrane is composed entirely of long-chain 1,2-diols [5], [6] and its cell wall is dominated by a protein rich in proline, glutamate, glycine, and alanine [7].

Since *T. roseum* was originally assigned to its own phylum, *Thermomicrobia* [8], we selected it for sequencing as a representative of a novel phylum. Subsequently, in 2004, *T. roseum* was reassigned to the phylum *Chloroflexi* based on phylogenetic analysis of 16S rRNA genes [9]. Here we report on the sequencing and analysis of the genome of *T. roseum* strain DSM 5159, the type strain for this species.

## Results and Discussion

### Genome characterization defines a chromosome and a megaplasmid

**Figure 1:**
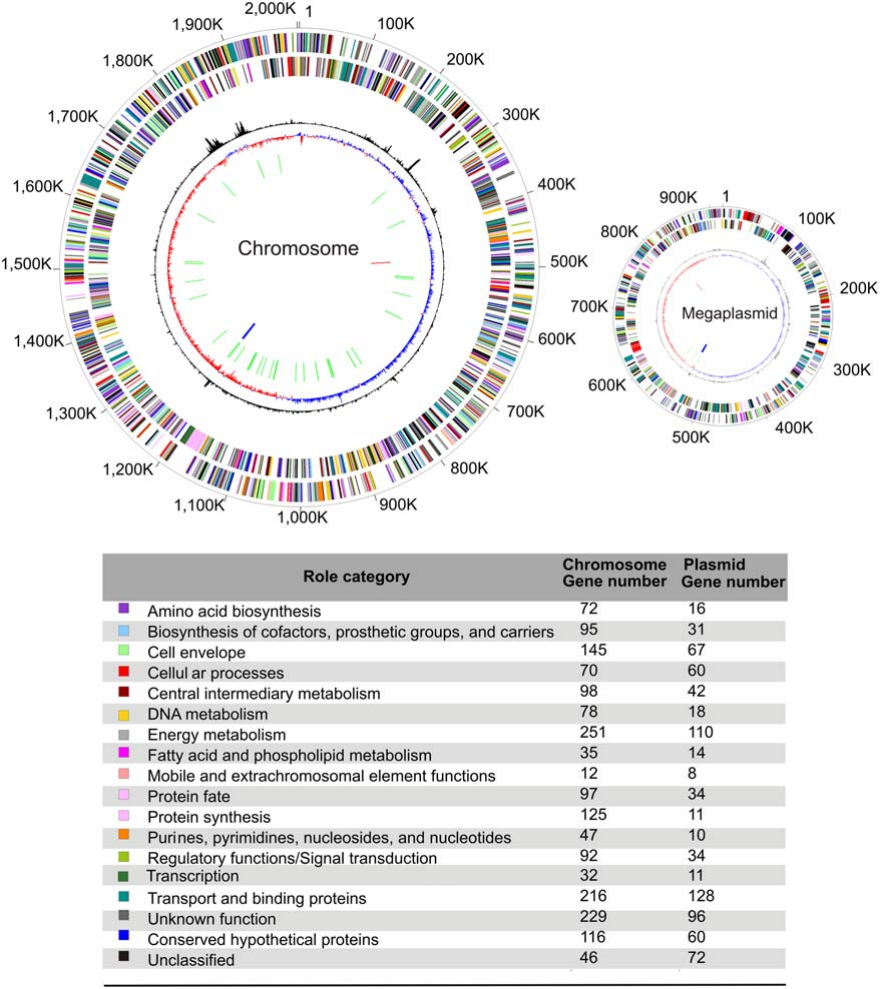
The chromosome and the megaplasmid of *T. roseum*. The circles display the following features, starting with the outermost circle: (1) forward strand genes; (2) reverse strand genes; (3) chi square deviation of local nucleotide composition from the genome average; (4) GC skew (blue bars represent positive values, red bars represent negative values); (5) tRNAs (green lines); (6) rRNAs (blue lines); (7) small RNAs (red lines). Gene color indicates the assigned role category. A gene can be included in the gene count for multiple role categories.

Full sequencing of the *T. roseum* genome revealed two circular elements (Table 1; Figure 1) of 2,006,217 bp and 919,596 bp, respectively. Both elements have high G+C content (Table 1), averaging 63.6% for the larger element and 65.7% for the smaller one. Likewise, both show a strongly mirrored GC skew pattern along the axes connecting their origin and terminus of replication. The shotgun sequence reads from both elements have average coverage depths of ∼9.7 suggesting that they are present in the same number of copies per cell.

**Table 1:** Genome features of *T. roseum*.

	*T. roseum* chromosome	*T. roseum* megaplasmid
**Sequence length (bp)**	2006217	919596
**G+C content (%)**	63.64	65.67
**G+C content of protein coding region (%)**	63.57	65.56
**G+C content of non-coding region (%)**	63.7	65.91
**Coding content (%)**	90.18	87.18
**Predicted protein coding sequences (CDSs)**	1925	937
**rRNAs**	3	3
**tRNAs**	46	3
**Small RNA genes**	1	1
**Average protein length**	313	285
**Average pI of proteins**	7.4	7.9
**Genes with functional role assignments (%)**	73.7	62.3

We refer to the larger element as a chromosome and the smaller one as a megaplasmid largely due to the differences in the array of predicted functions encoded by their respective genes. In particular, although both elements contain one rRNA operon, the megaplasmid contains only three of the 46 tRNAs and encodes few standard “housekeeping” proteins (e.g., ribosomal proteins, subunits of the RNA and DNA polymerases). Furthermore, phylogenetic analyses of the few homologs of housekeeping proteins encoded on the megaplasmid indicate that, unlike those encoded in the chromosome, they tend to not group with genes from other members of the *Chloroflexi* (see section on megaplasmid evolution below). Nevertheless, some important functions are encoded on the megaplasmid (see the section on flagella, below).

**Figure 2:**
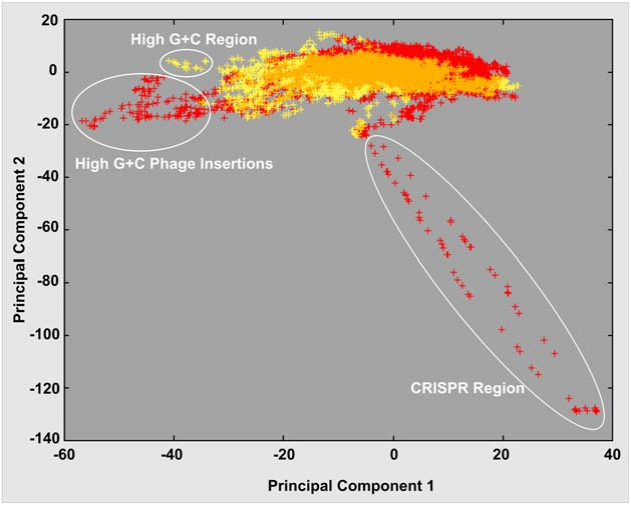
Analysis of nucleotide composition variations within the *T. roseum* chromosome and megaplasmid. Hexamer frequencies were extracted from 20,000 random 4 kb fragments from the whole genome and analyzed using CompostBin, a PCA-based method. The first two principal components of the data are plotted. Red indicates chromosome data; yellow indicates megaplasmid data; orange indicates overlaps between the two; circles indicate the three outlier regions.

The nucleotide composition of the chromosome was analyzed by both a chi square test of trinucleotides and the CompostBin principal component-based method (see Materials and Methods). These analyses revealed three regions that are markedly different in composition compared to the rest of the genome (Figures 1& 2). Two of these are relatively short segments (18 kb and 11 kb) of very high G+C content (73%) that are separated by a run of approximately 50 kb of relatively normal composition. These high G+C regions contain homologs of numerous genes that likely are either derived from phage such as phage domain protein trd_1586 and phage structural protein trd_1644, or encode phage-related activities (e.g., a peptidase trd_1587 and an N-acetylmuramoyl-L-alanine amidase trd_1647 thought to be used by phage to break links in bacterial cell walls). Therefore, we propose that these two regions of anomalous composition represent prophage relics. The third anomalous region was identified by the low complexity of its base sequence and by its relatively low G+C content (55%). It was found to contain clustered regularly interspaced short palindromic repeats (CRISPRs) which are implicated as a phage-resistance system [10], [11]. The megaplasmid also contains a 7.5 kb region of highly anomalous composition (71% G+C), but efforts to pinpoint its origin were inconclusive.

Genes for non-coding RNAs as well as putative protein coding regions were identified, and our summarized findings are presented in Table 1. Gene functions were predicted and genes were assigned to role categories using the automated system developed at The Institute for Genomic Research (TIGR) and applied to many previous genomes. To avoid bothersome repetition when discussing predicted capabilities, we often omit the term “predicted,” but they are predictions nevertheless. We do note whenever the predictions are either particularly robust or ambiguous.

### An important role for non-photosynthetic *T. roseum* in studying the evolution of photosynthesis in the phylum *Chloroflexi*

**Figure 3:**
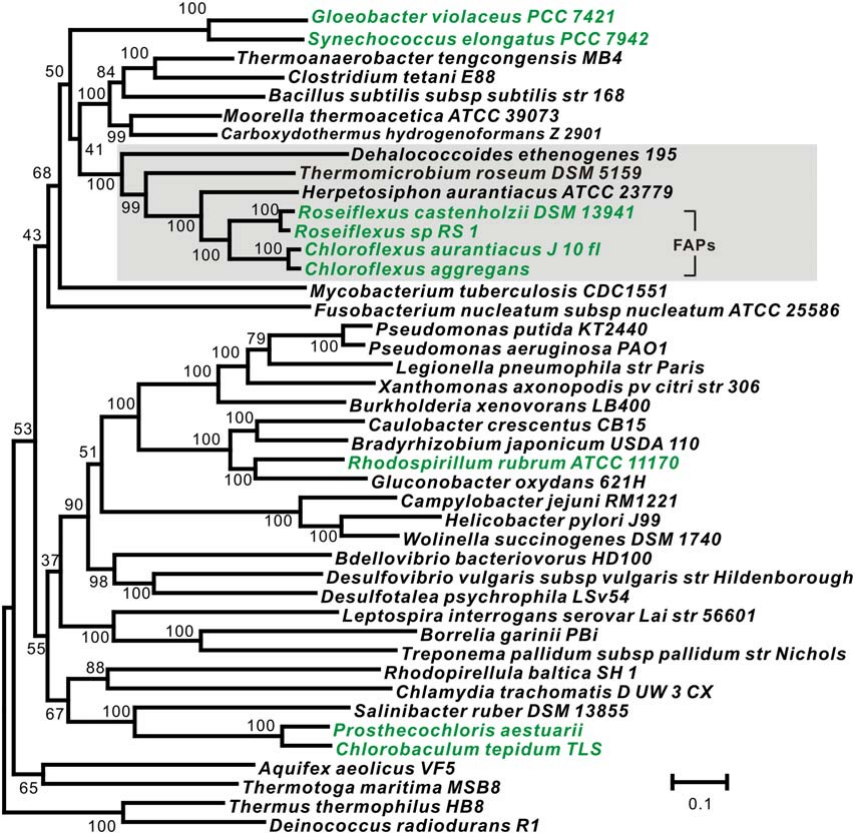
Maximum-likelihood tree of *Thermomicrobium roseum* and other bacterial species for which complete genomes are available. The tree was built from concatenated alignments of 31 housekeeping genes using the PHYML program. The bootstrap values are based on 100 replications. Organisms with photosynthetic capability are emphasized in green. Members from the phylum *Chloroflexi* are highlighted by the shaded box. Note – the organism *Chlorobaculum tepidum* used to be known as *Chlorobium tepidum*.

One of the main goals of our aTOL project was to use the sequenced genomes generated to elucidate the evolutionary relationships among the bacterial phyla. We selected *T. roseum* as a representative of the phylum *Thermomicrobia* [8]. After sequencing began, this phylum was formally merged with the phylum *Chloroflexi* based on phylogenetic analysis of 16S rRNA gene sequences [9]. Since there are multiple examples for which trees based solely on 16S rRNA gene sequences have proven to be misleading [12], [13], we re-evaluated the phylogenetic placement of this species using a genome-scale phylogenetic analysis. Our results support the placement of *T. roseum* in the phylum *Chloroflexi* (Figure 3).

On one hand, this was disappointing because it meant that we were not sequencing the first genome from a novel phylum. On the other hand, the fact that this species is not as isolated compared to other genomes makes its genome more valuable for certain comparative genomic analyses. Perhaps the most important potential comparative use for the *T. roseum* genome will be in elucidating the evolution of photosynthesis in the *Chloroflexi*. Although this organism is non-photosynthetic, it can be used to study the evolution of photosynthesis in this group much as non-pathogens are used to study the origin of pathogenicity in their close relatives and flightless dinosaurs are used to study the evolution of flight in birds.

The photosynthetic members of the *Chloroflexi* belong to the family *Chloroflexiaceae*. Previously referred to as the “green non-sulfur bacteria,” they are now known as the filamentous anoxygenic phototrophs (FAPs). The FAPs are a key group for deciphering the evolutionary history of photosynthesis because their photosynthetic reaction centers are similar to those of the “purple” proteobacteria [14] while their bacteriochlorophyll biosynthetic genes and light-harvesting chlorosomes are similar to those of the green sulfur bacteria and *Candidatus* Chloracidobacterium thermophilum [15]–[17]. Prior studies have suggested that this chimeric photosynthetic system resulted from horizontal transfer of genes encoding photosynthetic system components between phylogenetically distant groups [18]. However, the directions of transfer and the exact origins of the various components remain unresolved.

*T. roseum*, being one of the most closely related non-photosynthetic species to the FAPs to have its genome sequenced (Figure 3), serves as an important reference point for unraveling the evolution of photosynthesis in FAPs. We note that the only more closely related species to the FAPs than *T. roseum* for which a genome sequence is publically available is *H. aurantiacus*. Since there is no publication yet associated with the *H. aurantiacus* genome data, we are not carrying out a complete analysis of that genome here. In terms studying the evolution of photosynthesis, we sought to use the *T. roseum* genome to test one of the possible explanations for the origin of photosynthesis in the FAPs – that the FAPs co-opted genes that were present in an ancestor and that were involved in some process other than photosynthesis. If this occurred, then some of the genes involved in photosynthesis in FAPs might be closely related to genes found in non-photosynthetic *Chloroflexi* such as *T. roseum*.

To test for co-option, we built phylogenetic trees for all genes in the FAPs known to be directly involved in photosynthesis. We then searched these trees for instances in which a photosynthetic gene from a FAP grouped with genes from non-photosynthetic *Chloroflexi* rather than with genes from photosynthetic species in other phyla.

Of the 122 genes included, only one, an auracyanin homolog (trd_0373), meets the criteria for cooption from an ancestral function (Supplement Figure S1). Auracyanins are thought to be soluble donors that transfer electrons to the photosynthetic reaction center [19]. This suggests the possibility that the auracyanins used in photosynthesis were co-opted from another electron transport function. Other than this one example, we find no evidence supporting the theory that genes were co-opted for photosynthesis from other functions. Of course, it is possible that genes were co-opted from other functions and then that all of these genes were lost from non-photosynthetic species like *T. roseum*. However, it seems more likely that some other theory must explain the presence of photosynthesis in the FAPs. The two main possibilities that remain are acquisition of all the genes required for photosynthesis from other lineages after the *Chloroflexi* branch separated from *T. roseum* or the presence of photosynthesis in the ancestor of the whole phyla and then the loss of this process and all of the genes involved from non photosynthetic species.

We note that we also do not find any relics of the carbon fixation pathways of photosynthetic *Chloroflexi* in the non-photosynthetic ones. Both *T. roseum* and *D. ethenogenes* have traces of the Wood-Ljungdahl pathway [20]. In addition, *T. roseum* might encode genes for Calvin-Benson-Bassham cycle as well (as discussed below) [21]. All the carbon fixation pathways of the non-photosynthetic *Chloroflexi* members are quite different than the 3-hydroxypropionate pathways of the FAPs [22]. Thus it seems that carbon fixation pathways of the FAPs also did not arise by co-option from ancestral functions.

### Genomic insights into the synthesis and function of the atypical components of the *T. roseum* cell envelope

Another key goal of our aTOL project was to understand better the genomic basis of unique phenotypes found among novel phylogenetic groups. One of the most unusual known aspects of the biology of *T. roseum* is its cell envelope. Here we describe the genomic and experimental analyses we used to investigate these novel structures.

Compared to other bacteria, the cell wall of *T. roseum* contains only a small amount of peptidoglycan [7], but this small amount appears to be essential because *T. roseum* is sensitive to penicillin [4]. Galactosamine is substituted for glucosamine, the usual peptidoglycan building block [7]. A standard peptidoglycan synthesis pathway is encoded in the genome. Five transpeptidases are involved in the cross-linking of the peptidoglycan (trd_0058, trd_1373, trd_1927, trd_0835, trd_1968), including one bifunctional protein that also has a transglycosylase domain (trd_1968).

Also unusual, the *T. roseum* cell wall is dominated by proteins, primarily by a single 75,000 kDa protein that is rich in glycine, proline, glutamate, and alanine. No predicted protein in the genome matches this profile, suggesting that this protein undergoes significant modification after translation. We think it likely that some cell wall proteins are cross-linked to the peptidoglycan. This could also explain the presence of ornithine in some of the protein fractions [7] because the acid hydrolysis could release ornithine that is frequently found in petidoglycans [23].

The cytoplasmic membrane of *T. roseum* is also novel; it is made of a series of long chain 1,2-diols instead of the glycerolipids found in most bacteria [5]. A 1,2-diol is structurally similar to a glycerolipid except that the third carbon is linked to a fatty acyl group instead of being linked to a hydroxyl group that is esterified with a fatty acid. Notably, growing *T. roseum* at higher temperatures results in fewer branched chained diols and a slightly greater diol chain length [6]. We note that the absence of a homolog of the *plsB* gene (glycerol-3-phosphate acyltransferase) suggests that the normal glycerol-phospholipid synthesis pathway is blocked, and thus 1,2-diols must substitute for glycerolipids as the membrane building blocks for *T. roseum*. Fatty acids can be reduced and hydrolyzed to produce 1,2-diols, but the details of the processes remains to be clarified.

### Characterization of glycosylated carotenoids in *T. roseum* and their possible role in maintaining cell membrane integrity

The carotenoids which give *T. roseum* colonies their pinkish-red color are another key component of their novel cell membrane. The structures of the pigments and their synthetic pathways have not previously been characterized in detail. We present here the results of our genome analysis and high performance liquid chromatography (HPLC) studies investigating these carotenoids and their synthetic pathways.

**Figure 4:**
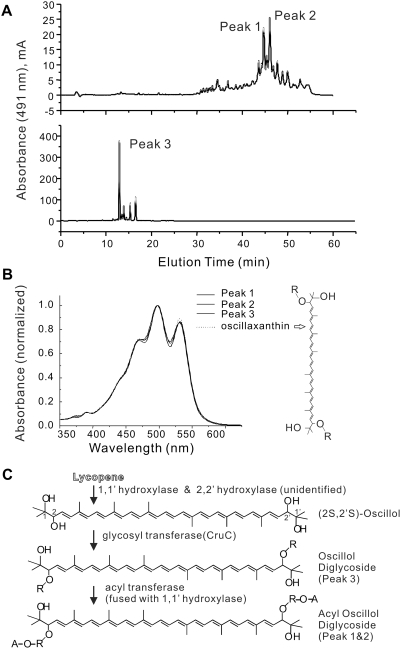
Carotenoid modification in *T. roseum*. A. Reverse phase HPLC analysis of *T. roseum* pigments revealed two types of carotenoid compounds: extremely hydrophobic pigments from crude extracts (top panel, peaks 1 & 2) and a polar glycoside released by saponification (bottom panel, peak 3). The glycoside is the major form of carotenoid in *T. roseum*. Its HPLC retention time was similar to that of the authentic oscillaxanthin (oscillol-2,2′-difucoside) from the cyanobacterium *Gloeobacter violaceus* PCC7421 (see Figure 4B for the molecular structure). B. Comparison of the in-line spectra of compounds from peak 1, 2 & 3 (Figure 4A) with that of the oscillaxanthin from *Gloeobacter violaceus* PCC 7421 indicates that the major *T. roseum* carotenoid core is oscillol diglycoside. The structure of oscillaxanthin is shown to the right. The sugar moieties in oscillaxanthin are fucose; the identity and exact positions of the glycosyl moieties in the *T. roseum* oscillol diglycoside are yet to be determined. C. Carotenoid modification pathway in *T. roseum*. Step 1: The introduction of two hydroxyl groups at the ends of lycopene to form (2S, 2′S)-oscillol. The absorbance spectra of *T. roseum* pigments (Figure 4B) indicate that 1,1′ and 2,2′ positions have all been hydroxylated. We found a 1,1′ hydroxylase in the genome; the gene(s) encoding the 2,2′ hydroxylation function are yet to be identified. Step 2: A glycosyl transferase (CruC) adds a sugar moiety (R) to both ends of (2S,2′S)-oscillol to produce oscillol diglycoside, the dominant carotenoid (Figure 4A, peak 3). The nature of the sugar moieties are unknown. Although the figure shows a 2,2′-oscillol diglycoside, 1,1′-oscillol diglycoside is another possibility at this step. Step 3: The acyltransferase domain of the 1,1′ hydroxylase adds acyl chains (A) to the sugar moieties (R), thus producing the extremely hydrophobic pigments seen in the crude extracts (Figure 4A, peak 1&2).

Based on the absorbance spectra of crude extracts, the carotenoids of *T. roseum* were first thought to be derivatives of torulene or 3,4-dehydrolycopene [4]. We think that this identification was incorrect, an error likely due to the poor pigment yields obtained by extracting the cells with acetone/methanol. Our HPLC analyses of such extracts suggested that a complex mixture of very hydrophobic compounds was present (Figure 4A, upper panel). However, after saponification, pigment yields were much higher (Figure 4A, lower panel) with the major carotenoid being very polar and displaying a retention time similar to that of authentic oscillaxanthin (oscillol-2,2′-difucoside) from the cyanobacterium *Gloeobacter violaceus* strain PCC7421 [24]. The visible absorption spectra of both the acylated compounds and the glycoside liberated by saponification were identical to that of oscillaxanthin (Figure 4B, 4C). They are also consistent with those of acylic carotenoids with 13 conjugated double bonds. The complexity of the elution profile and the extremely hydrophobic nature of the pigments prior to saponification prompt us to believe that the major pigments of *T. roseum* have modifications that are similar to those on the ψ end of salinixanthin, a carotenoid glycosyl ester produced by *Salinibacter ruber* [25]. If the *Salinibacter ruber* type of carotenoid acylation and glycosylation takes place in *T. roseum*, we believe the major carotenoids of *T. roseum* are acylated derivatives of an oscillol-di-glycoside; however, the identity and attachment position (*i.e.*, the 1,1′ or 2,2′ hydroxyls) of the glycosyl moieties have not yet been determined

The genome sequencing data support and further explain the biochemical analyses. A single gene cluster (trd_1158 to trd_1167) on the *T. roseum* chromosome encodes most of the genes predicted to be related to carotenoid biosynthesis and modification. This cluster includes a gene encoding phytoene synthase (*crtB*; trd_1159), a phytoene dehydrogenase (*crtI*; trd_1163), a 1,1′ hydroxylase (trd_1162) that adds a hydroxyl group to one or both ends of lycopene [26], and a group II glycosyl transferase (*cruC*; trd_1161) that adds a glycosyl moiety to the hydroxyl groups at the ends of carotenoid molecules [27] (Figure 4C).

Interestingly, the 1,1′ hydroxylase (trd_1162) has an acyltransferase domain at the C-terminus of the predicted protein that is presumably responsible for acylation of the glycosyl residues introduced by CruC (Figure 4C, Steps 2 & 3), the dual functions of this *T. roseum* protein are carried out by two different enzymes in *Salinibacter ruber*: the 1,1′ hydroxylase (*cruF*) and acyltranferase (*cruD*). Genes encoding apparent orthologs for each of the other enzymatic functions for the ψ-end modifications are also found in the genome of *Salinibacter ruber* [28]. In addition, this gene cluster encodes two conserved hypothetical proteins (trd-1160, trd_1167) and a paralog of glycolate oxidase (trd_1165). One or more of these genes might be responsible for the proposed introduction of the 2,2′ hydroxyl groups (Figure 4C, Step 1). No homologs were found to any of the four known classes of lycopene cyclases [29], a finding that is consistent with the chemical analyses that identified only linear carotenoids in this organism.

The genome sequence and biochemical analyses show that *T. roseum* can produce a complex mixture of dipolar carotenoids. Because it is not phototrophic, these carotenoids probably serve as biological antioxidants that protect the cells against singlet oxygen and free radicals [30], [31]. It has also been demonstrated that carotenoids can significantly modify the physical properties of the lipid phase of membranes [32]. Specifically, they decrease membrane fluidity, increase the order of alkyl chains, and increase the hydrophobicity of the membrane interior. Such effects are strongest for dipolar carotenoids like those found in relatively great abundance in *T. roseum* [33]. Given these properties and the absence of various glycerol-derived phospholipids, such as cardiolipin and phosphatidylethanolamine, in *T. roseum*, it is probable that carotenoids modified by glycosylation and acylation at both ends of the C-40 backbone play an important role in membrane stabilization in this thermophile (Figure 4C).

### Chemoheterotrophic growth and nutrient requirements

As part of our aTOL project, we are characterizing the growth requirements for each selected species so that these organisms can be better utilized in the lab for experimental studies. Thus, we initially focused on the growth and nutrient requirements for *T. roseum*.

Given its well-documented ability to grow chemoheterotrophically [4], the *T. roseum* genome not surprisingly encodes complete pathways for glycolysis/gluconeogenesis, the oxidative pentose phosphate pathway, and the TCA cycle, as well as the components of an electron transport chain coupled with oxidative phosphorylation. Likewise, we identified complete pathways for the biosynthesis of pyrimidine/purine nucleotides, amino acids, and many vitamins and other cofactors. However, the mechanism by which nitrogen is acquired for biosynthetic pathways is unclear. Experimental studies have shown that *T. roseum* required the addition of glutamate to grow if nitrate was the source of inorganic nitrogen [4]. Genes for the reduction of nitrate to nitrite and for the assimilation of ammonia (*gdhA*, trd_1476) are present in the genome. However, we could not identify any genes encoding enzymes known to carry out the conversion of nitrite into ammonia. Specifically, we could detect neither an NADH-dependent nor a formate-dependent nitrite reductase—the enzymes required for the two standard pathways [34], [35].

Based on our genome analysis, we predict that *T. roseum* can synthesize a wide variety of water-soluble cofactors in the vitamin B family, including thiamine, riboflavin, pyridine nucleotides, pyridoxine, folate, ubiquinone, menaquinone, lipoate, heme, coenzyme F420, and molybdopterin. There are, however, some potentially important exceptions. Although most of a pathway for pantothenate synthesis is present, no ketopantoate reductase (*panE*) could be identified. Since a complete pathway for synthesizing coenzyme A from pantothenate is present, it seems likely that the pantothenate pathway is complete and, furthermore, that T. *roseum* can synthesize coenzyme A. We predict that *T. roseum* cannot synthesize vitamin B_12_, but we also note that homologs of enzymes that carry out two of the B_12_ synthesis steps in many species (corrin-porphyrin methylase trd_0852 and corrinoid adenosyltransferase trd_0805) are present. Apparently, *T. roseum* lacks the ability to synthesize glutathione, as both glutamate-cysteine ligase and glutathione synthetase are missing from its genome.

A key component of understanding the growth requirements for an organism lies in its capabilities for transporting molecules in and out of the cell. Because a large fraction of the genome (268 genes, 9.7% ORFs) is dedicated to membrane transport functions, *T. roseum* exhibits a very robust transporter system, largely due to the great expansion of importers for organic nutrients like sugars, amino acids and peptides. 104 transporter genes are encoded on the megaplasmid (12% of ORFs).

Of the predicted transport proteins, the multiple component ABC superfamily transporters are predominant (168 genes). Transporters in this family utilize ATP hydrolysis as the primary source of energy to drive the transport process. They are involved in the uptake of a large array of organic nutrients and inorganic compounds, as well as the export of iron compounds, peptides and multi-drugs. For example, *T. roseum* encodes 14 complete amino acid and peptide importers, 8 sugar importers and 4 nitrate importers. Previous studies show that photosynthetic organisms with the ability to synthesize an ATP pool via photosynthesis, like *Synechococcus elongatus* and *Gloeobacter violaceus* strain PCC7421 encodes relatively higher percent ABC family transporters compared to other organisms [36]. The distribution pattern of ABC transporters in *T. roseum* shares the similar feature as those photosynthetic organisms. One of the possible explanations could be that *T. roseum* can utilize CO and H_2_ as energy source and generate a large ATP pool (see later paragraphs for details).

*T. roseum* also encodes a large array of secondary-type transporters (76 genes), which utilize ion or solute electrochemical gradients, such as the proton/sodium motive force, to drive the transport process. Two families are relatively expanded compared to other *Chloroflexi* species: the MFS family transporter for drug efflux (23 genes) and sugar uptake (2 genes), and the APC family amino acid and ethanolamine importers (5 genes). *T. roseum* encodes a CPA3 family multi-component monovalent cation/proton antiporter (trd_A0369 to trd_A0375). Studies in alkaliphilic *Bacillus halodurans* C-125 shows that this transporter system is important for pH homeostasis and alkali tolerance [37]. It catalyzes efflux of cytoplasmic sodium, potassium or lithium ions in exchange for external hydrogen ions (protons). A multifunctional complex could provide synergies such as the presentation of a large protein surface area on the external membrane surface, which enhance proton capture and the monovalent cation/proton antiport function that supports alkali resistance [38]. The presence of this transporter system in *T. roseum* supports its adaptation to the alkaline hot spring environment.

### Alternatives to heterotrophic growth

As discussed above, *T. roseum* is known to grow as a chemoheterotroph [4]. However, our analysis of the genome suggested the potential for other nutritional modes as well. In the following sections, we describe the bioinformatic and experimental evidence supporting two other possibilities: chemotrophic growth using CO or H_2_ as an energy source and autotrophy via the fixation of CO_2_.

#### Genome analysis and experimental tests supporting aerobic CO oxidation

**Figure 5:**
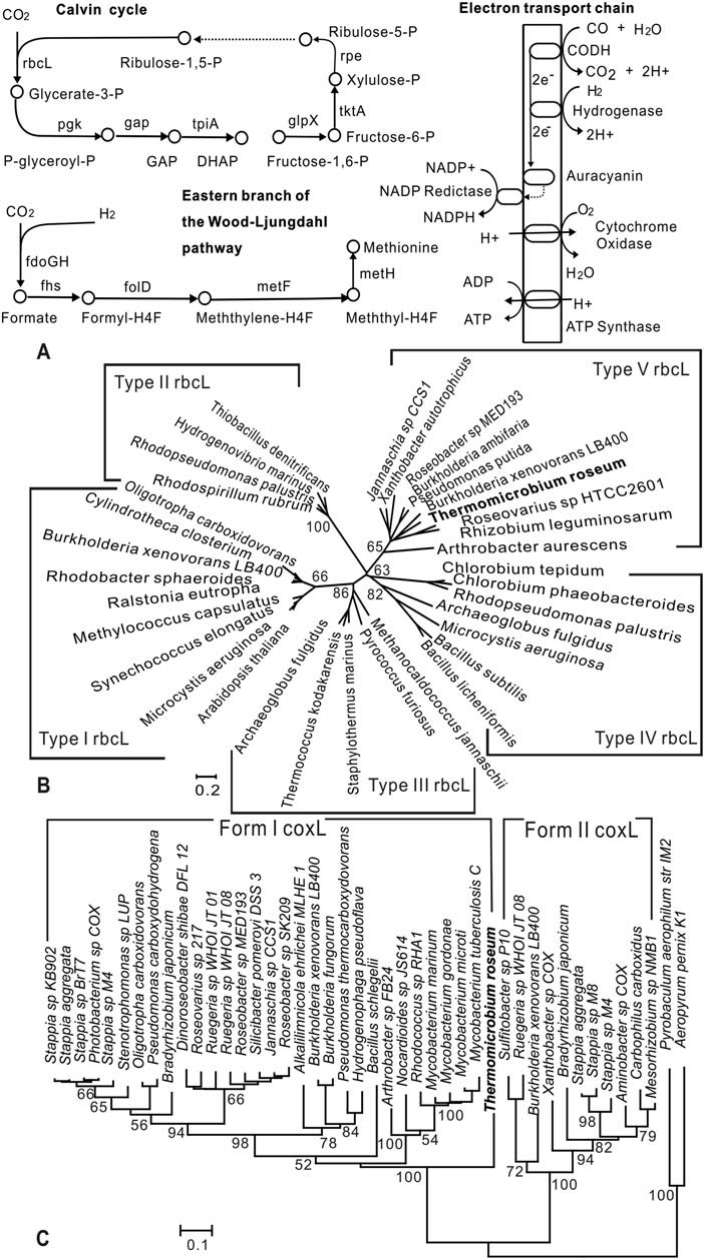
CO oxidation and CO_2_ fixation in *T. roseum*. A. Two pathways for carbon dioxide fixation and an electronic transport chain for carbon monoxide/hydrogen oxidation in *T. roseum*. B. Phylogenetic tree for the catalytic domain of Types I-V ribulose bisphosphate carboxylase large subunit RbcL). The tree is based on alignments generated using Pfam domain PF00016. C. Phylogenetic tree for the molybdopterin-binding domain of the aerobic CO dehydrogenase CoxL. The tree is based on alignments generated using Pfam domain PF02738.

The possibility that *T. roseum* utilizes carbon monoxide (CO) as an electron source was suggested by the presence of genes encoding homologs of the three subunits of a molybdopterin-containing carbon monoxide dehydrogenase (Mo-CODH trd_A0564-6) [39]. There are two known forms of Mo-CODH: form I oxidizes CO, whereas the function of form II remains uncertain [39]. Phylogenetic analysis identified the *T. roseum* gene as form I (Figure 5C). The genome also encodes a molybdate ABC transporter (*modAB*: trd_A0234-5) and a set of molybdenum cofactor biosynthesis proteins (*moaABCDE*: trd_1041, trd_1044, trd_1078, trd_1708), both of which are needed to provide the required molybdopterin cofactors.

**Figure 6:**
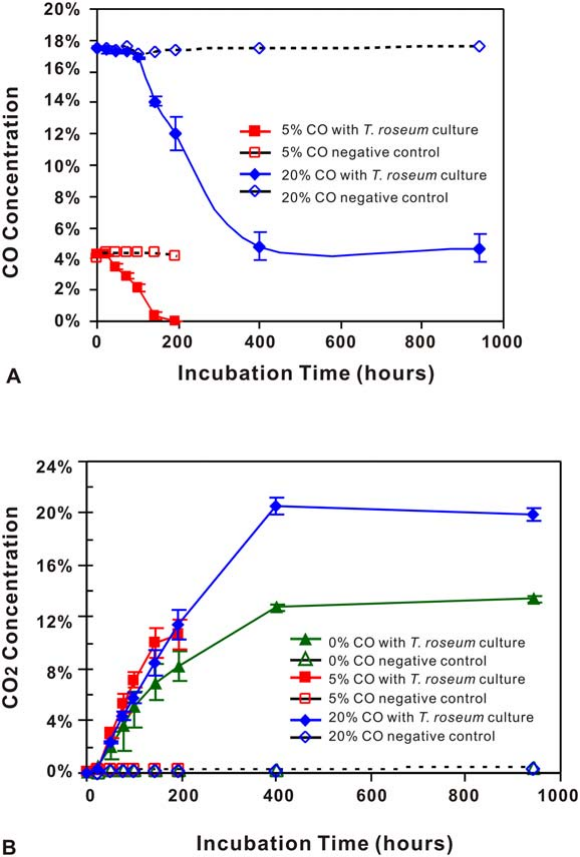
CO consumption and CO_2_ production by *T. roseum* cultures. A. CO concentration in headspace plotted against incubation time. For the 20% CO experiment: (a) O_2_ concentration (not shown) had decreased to below detection level at 400 hours; and (b) the onset of CO consumption was delayed by roughly 75 hours as compared with the 5% CO experiment. B. CO_2_ concentration in headspace plotted against incubation time. For the 20% CO experiment, O_2_ concentration (not shown) had decreased to below detection level at 400 hrs, whereas in the 0% CO control, it fell from an initial level of 20% down to about 0.5%. Initial CO_2_ production appeared to be highest in the 5% CO experiment (which had an earlier onset of CO oxidation), followed by the 20% CO experiment. Production of CO_2_ lagged in the *T. roseum* culture with 0% CO and plateaued at a lower level.

Based on these findings, we decided to test directly for CO utilization. Experiments showed that when CO is provided, *T. roseum* grows and oxidizes CO to produce CO_2_ (Figure 6). When the initial CO level is low (5%), all the CO is utilized; when more CO is provided (20%), a residual amount of CO remains (Figure 6A) due, we think, to limiting amounts of oxygen.

Aerobic CO oxidation is found only in a few groups of bacteria, specifically in many *Actinobacteria* and *Proteobacteria* and in at least one Firmicute [39]. Thus, its presence in a member of the *Chloroflexi* is relatively novel. Phylogenetic analysis did not provide any convincing evidence for gene transfer for any of the genes involved (data not shown). This presents a bit of a conundrum. The last common ancestor of the phyla in which this capability has been found likely predates the oxygenation of the atmosphere [40]. Thus, either (a) the ancestral process utilized something other than oxygen as the electron acceptor and subsequently each lineage separately evolved the means to utilize oxygen, or (b) the original process evolved in one phylum after oxygenation of the atmosphere and then moved by lateral transfer events to the other lineages.

#### Genomic evidence for use of H_2_ as an energy source

The possibility of using H_2_ as another source of high energy electrons is indicated by the presence in *T. roseum* of a *hox* gene cluster encoding the apparatus for utilizing hydrogen gas (H_2_) (trd_1863-78)[41]. This cluster encodes both the large (HoxL trd_1877) and small (HoxS trd_1878) subunits of a membrane-bound NiFe-hydrogenase involved in H_2_ uptake, hydrogenase expression-formation proteins (HypDE trd_1866-7), a maturation protein (HypF trd_1863), an assembly chaperone (HypC trd_1869), and a nickel-incorporation protein (HypA trd_1871). We note that HoxS and HoxL group in phylogenetic trees within a clade of proteins from actinobacteria, thus suggesting that the hox cluster may have been acquired by gene transfer.

#### Electron transport when using CO or H_2_ as energy sources

In aerobic organisms such as *T. roseum*, energy from the oxidation of CO or H_2_ is usually captured by some type of electron transport chain. There are two likely acceptors for the electrons provided by either Mo-CODH or NiFe-hydrogenase. One is oxygen, which, as discussed above, may be required for CO oxidation. This pathway would likely utilize a cytochrome oxidase, in which case the cytochrome oxidase must tolerate CO. Electron transport to oxygen might be used to generate a proton gradient which could, in turn, drive ATP generation via ATP synthase.

Alternatively, electrons could be channeled to NADP^+^ to generate NADPH via the novel NADP^+^ reductase encoded in the genome (trd_A0924). This predicted reductase contains not only a 2Fe-2S iron-sulfur cluster binding domain and an oxidoreductase FAD-binding domain, but also the only bacterial ferredoxin-NADP^+^ reductase NADP^+^ binding domain found in this genome.

The exact path by which electrons travel from CO or H_2_ to the final electron acceptors in *T. roseum* remains unclear. The auracyanin (trd_0373) discussed earlier in relation to the evolution of photosynthesis might play a role. Whatever the path, we predict that the CO and H_2_ oxidation pathways could serve as sources of both ATP and NADPH.

#### Potential for carbon fixation

In some species, ATP and NADPH generated from CO and H_2_ are used only as supplemental energy sources [39], while in others these energy-rich molecules are used for carbon fixation—a strategy termed *chemolithoautotrophy* because the energy source is chemical and the electron source is inorganic. Therefore, we examined the genome of *T. roseum* to assess its potential for carbon fixation. We found homologs for some genes involved in the CO_2_ fixation pathway used by some of the photosynthetic *Chloroflexi* (the 3-hydroxypropionate cycle). However, many are missing and those that are present appear to be paralogs, not orthologs. Thus, we conclude that this pathway is not likely to be present in *T. roseum*.

However, the genome does encode portions of two alternative CO_2_ fixation pathways. The first of these involves a variant of the Wood-Ljungdahl pathway (also known as the acetyl-CoA pathway). In the standard Wood-Ljungdahl pathway, CO_2_ is reduced with the formation of acetyl CoA. Due to the absence of the CO dehydrogenase/acetyl-CoA synthase (CODH/ACS), the Wood-Ljungdahl pathway is not complete. However, the “Eastern” portion [42] is present (Figure 5A) and would allow CO_2_ assimilation via methionine rather than via acetyl-CoA.

The second possible carbon fixation pathway is the Calvin Benson Bassham (CBB) cycle (Figure 5A). The genome encodes candidates for many of the steps of this cycle including a homolog of RbcL (trd_0132), the large subunit of ribulose 1,5-bisphosphate carboxylase/oxygenase (rubisco), although no gene for the Rubisco small subunit RbcS has been found in the genome. However, the absence of enzymes such as sedoheptulose 1,7-biphosphate aldolase, sedoheptulose bisphosphatase, and fructose 1,6-biphosphate aldolase indicates that *T. roseum* may not be able to regenerate ribulose 5-phosphate. The observation that *T. roseum* can grow on a three-carbon compound such as glycerol [4] suggests that a pentose regeneration process catalyzed by other aldolases and phosphatases may exist in *T. roseum*; however, we have been unable to identify a potential ribulose 5-phosphate kinase (PRK; phosphoribulokinase), an enzyme without which the CBB cycle would be incomplete.

If this is indeed the case, it would not be the first time an organism with an incomplete CBB cycle has been found to have an RbcL homolog that is used for some other purpose. RbcL homologs have been divided into four types based on comparative studies and phylogenetic analysis. Types I and II function as the “classic” Rubisco in the CBB cycle [43]. Type III forms are found only in archaea where they function as Rubisco in AMP metabolism. Type IV forms were first detected through genome sequencing [44]–[47] and have now been found in a variety of bacteria and archaea. Those that have been tested do not have detectible Rubisco activity [48]. The one from *B. subtilis* is a 2,3-diketo-5-methylthiopentyl-1-phosphate enolase that is involved in a methionine salvage pathway [49], [50].

Our phylogenetic analysis placed *the* RbcL homolog of *T. roseum* with sequences from proteobacteria of diverse biology, such as the aerobic anoxygenic phototrophic *Jannaschia sp. CCS1* and the nitrogen-fixer *Rhizobium leguminosarum*, (Figure 5B). Some of these were previously classified as type IV-NonPhoto because they were found in nonphototrophic bacteria [48]. However, our analysis indicates that this group, including the *T. roseum* homolog, is distinct. We propose it be designated as type V.

The functions of the type V RbcL homologs, including that of *T. roseum*, are unknown. Although some type V RbcLs are involved in the methionine salvage pathway, this is highly unlikely for the *T. roseum* RbcL because other genes for that pathway are not present in its genome. However, analysis of sequence and structural alignments suggests that the *T. roseum* protein has good potential to be a true Rubisco. With the exception of the beta-hairpin, most of the secondary structures present in typical photosynthetic rubisco large subunits are preserved in the *T. roseum* RbcL. Furthermore, the *T. roseum* RbcL has the loop CD structure that is found exclusively in type IV and V RbcLs [48]. When comparing the type IV and type V RbcLs, the protein sequences at the active sites of type V RbcLs were found to be most similar to those of the type I and II RbcLs that function as true rubiscos [48]. Specifically, 18 out of 19 of the amino acids at the active site in the *T. roseum* RbcL are identical to those in the type I and II forms.

### Responding to environmental cues

#### Signal Recognition and Transduction

3.5% of the genome of *T. roseum* is dedicated to signal transduction systems that equip this bacterium to sense and respond to environmental signals. It has been suggested that one-component systems in which one protein provides both the input sensor and the response regulator functions are the dominant signal transduction systems in bacteria because of their extraordinary combinatorial diversity [51]. Indeed, the largest group of signaling-related genes in *T. roseum* is the 64 genes for “one-component systems.” There are also 33 genes involved in two-component signaling systems, systems in which the sensor and response regulators are located in different proteins. Typically, the sensor protein is a histidine kinase, which transmits the signal to a response regulator by protein phosphorylation. *T. roseum* encodes 9 histidine kinases, 16 response regulators, and 8 chemotaxis-related proteins.

The ability to predict the input signals and output responses for these signaling systems provides some insight into what this organism responds to and how such responses are carried out. For example, DNA binding domains are by far the most abundant response domains found in both types of signaling system, being identified in 12 two-component and 39 one-component system proteins. This suggests that transcription regulation is used extensively to respond to environmental cues. Other responses can be predicted from the protein domains present, which include di-guanylate cyclase, hydrolase, phosphatase and protein kinase domains. Only a few predictions of the input signals sensed by these systems could be made confidently, and most of these cases involved responses to the binding of small effectors.

#### Chemotaxis and flagellum-related genes

Chemotaxis is a process by which bacteria move in response to chemical gradients in their environment [52]. Almost one third of the two-component system proteins (10 out of 33) in *T. roseum* are predicted to be involved in chemotaxis. The predicted proteins include a histidine kinase (CheA trd_A0215), a response regulator (CheY trd_A0211), a methyl transferase (CheR trd_A0210), and seven methyl-accepting chemotaxis proteins (MCPs). This is an intriguing finding in that *T. roseum* is not known to be motile [4].

**Figure 7:**
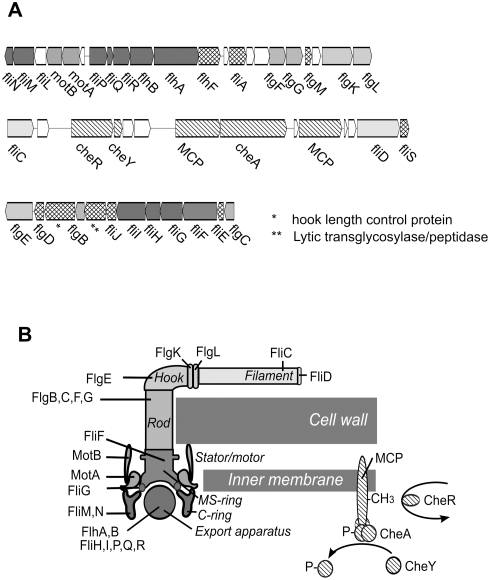
The megaplasmid encoded flagellar system in *T. roseum*. A. The three clusters of genes encoding flagellum-related and chemotaxis-related proteins located on the megaplasmid. B. The flagellar structures and related chemotaxis apparatus encoded in the *T. roseum* genome.

Likewise, it was surprising to find genes for synthesizing, assembling, and regulating a flagellum. Many of these genes are located in three gene clusters on the megaplasmid (Figure 7A). Identified genes encode all of the typical components of a flagellum including the cap (trd_A0220), filament (flagellin trd_A0208), hook (trd_A0642), rod (trd_A0645, trd_A0653, trd_A0044-5), MS-ring (trd_A0651), C-ring (trd_A0028-9), motor (trd_A0031-2), and type III flagellar export apparatus (trd_A0037-8, trd_A0648-9, trd_A0034-6) (Figure 7B) [53]. Genes related to flagellum assembly are also present, including the transcription-level regulator (*flgM* trd_0084) and an RNA polymerase sigma factor (*fliA* trd_A0041), the flagellin chaperon (*fliS* trd_A0221), the hook capping protein (*flgD* trd_A0643), and the flagella number regulator (*flhF* trd_A0039). The required ability to penetrate the cell wall also appears to be provided by a lytic transglycosylase/peptidase fusion protein (trd_A0646) that is located in one of the flagellar gene clusters. In addition, as discussed above, *T. roseum* possesses key genes for chemotaxis signal transduction.

This genetic repertoire suggests that *T. roseum* has, or recently had, the potential for making a flagellum and for chemotaxis. However, exactly how such a flagellum would attach and move the cell remains ambiguous. In most Gram-negative bacteria, the flagellum is anchored across the cell wall and outer membrane via the P-ring and L-ring structures [53]. Although *T. roseum* has the typical layered cell wall of a Gram-negative bacterium, including a structured outermost layer with a regular repeating unit [4], genes encoding P-ring and L-ring structures appear to be absent (Figure 7B). However, these two rings are also missing in organisms that do not have the typical Gram-negative flagellar attachment system, e.g., in spirochetes in which the flagella rotate between the outer membrane sheath and cell cylinder, and in Gram-positive bacteria which lack the outer membrane [54], [55]. It seems unlikely that the rod-shaped *T. roseum* could use a spirochete-like mechanism. In addition, although there are four genes that may be related to lipopolysaccharide biosynthesis, it appears unlikely that *T. roseum* has a standard Gram-negative structure with an outer membrane because it lacks the genes for outer membrane proteins, porins, periplasmic chaperones, and the YaeT/YfiO/YgL/NlB complex [56] makes. Another possibility is that the outer layer of *T. roseum* is part of its atypical cell wall, in which case its flagellar structure could be expected to resemble that seen in Gram-positive bacteria [7].

Even though flagella have not been reported for *T. roseum*, we suggest that *T. roseum* has the *potential* to make a functional flagellum. It is possible that one or more of the key genes have subtle sequence changes that render them inactive, but we have not observed any such mutations. Clearly, more research is needed in order to determine the structure of the outer layer of *T. roseum* cells, and more growth conditions will likely have to be explored in order to prompt *T. roseum* to demonstrate its flagellar and chemotaxic potentials.

### Flagellum-related genes all located on the megaplasmid

The evolution of the multi-component flagellar apparatus has been a subject of great interest. None of the other species in the *Chloroflexi* group whose genome has been sequenced encode any flagellar structural components. The chemotaxis signal transduction proteins (e.g., *cheR*, *cheY*, *cheA*, and *mcp*) identified in some of them (*e.g.*, *C. aggregans*, *C. aurantiacus*, and *H. aurantiacus*) [57] probably function in conjunction with their filamentous gliding motility [58]. *D. ethenogenes*, which lacks the flagellar structural protein genes, has genes that encode the structural and assembly proteins for a twitching motility and type IV pilus system [59].

We were particularly interested in how *T. roseum* came to possess all of the genes for making a flagellum when most other members of the *Chloroflexi* do not. Phylogenetic analysis reveals that neither *cheR* nor *cheY* in *T. roseum* branches with other *Chloroflexi* homologs, while *cheA* is the deepest branch in a *Chloroflexi* clade with a very long branch length. The cell motility related signal transduction proteins in *T. roseum* are only distantly related to those of the other *Chloroflexi*, thus making it unlikely that the genes were present in a common ancestor or that they were acquired by lateral transfer within the *Chloroflexi*.

However, there is one very striking finding that suggests *T. roseum* may have acquired its entire flagellar apparatus by gene transfer: except for several methyl-accepting chemotaxis proteins, all of the flagellum-related genes are located on the megaplasmid (Figure 7A). Such localization of all flagellum-associated genes on a potentially mobile element has not been reported previously in either the Bacteria or the Archaea.

The origin of the flagellar genes in *T. roseum* is thus related to the origin of the megaplasmid. Three lines of evidence suggest that this megaplasmid was acquired after the *T. roseum* lineage diverged from the lineages of all other *Chloroflexi* for which genomes are available. Firstly, only one other species in the *Chloroflexi* is known to contain a megaplasmid, that being *Herpetosiphon aurantiacus* (Bryant, unpublished). We did not detect any evidence for a close relationship between these two megaplasmids. Secondly, as discussed above, phylogenetic analysis of the few homologs of housekeeping proteins encoded on the megaplasmid indicates that, unlike the housekeeping proteins encoded in the chromosome, these proteins tend to not group with genes from other members of the *Chloroflexi*. Thirdly, CompostBin nucleotide composition analysis (Materials and Methods) shows that despite significant overlap between the two genetic elements, one can still clearly detect differences between their patterns of nucleotide composition (Figure 2).

It has not escaped our notice that the presence of all the flagellar components on a mobile element suggests a simple and rapid means by which organisms could acquire motility.

### Environment genomics analysis

Since its isolation from Toadstool Spring in Yellowstone National Park [4], *T. roseum* has not been convincingly identified in any other environment. Recently, metagenomic sequence data was reported from microbial mats of two other hot springs in Yellowstone: Octopus Spring and Mushroom Spring (which is immediately adjacent to Toadstool Spring) [60]. These hot springs are similar in many ways to Toadstool with relatively stable bacterial and archaeal communities with phototrophic upper layers (which are mainly composed of cyanobacteria and FAPs) and heterotrophic deeper layers [60]. In the metagenomic data for both springs we have identified sequences that come from organisms that appear to be very closely related to the *T. roseum* strain we sequenced (Supplement Figure S2). The observation that the sequences that are similar to the *T. roseum* genome are dispersed over the entire genome suggests that an organism closely related to *T. roseum DSM5159* is present in these habitats (instead of particular DNA segments recently acquired by other organisms from *T. roseum*). Previous work had suggested that *T. roseum* was present in Octopus Spring (because lipids like those from *T. roseum* had been identified there) but the closest 16S rRNA sequence from the spring only shared 75.4% similarity with *T. roseum* [61].

We note that the sequences that are from close relatives of *T. roseum* in the metagenomic data are relatively rare in the metagenomic data sets (Figure S2 A). This suggests that at least for these samples, the *T. roseum-like* organisms have a relatively low abundance (the unevenness in the number of the sequence reads from different depth samples might contribute to the large variation in *T. roseum*-like sequences in the different samples (Figure S2 A)). Nevertheless, *T. roseum*–like sequences are seen in slices sampled at as deep as 5 mm from the microbial mats – a finding that is consistent with our prediction that the strain we sequenced has a dependency on the availability of oxygen instead of light as revealed by our sequencing efforts.

Although the high levels of sequence similarity to the metagenomic reads suggest that a relative of the strain whose genome we sequenced is present in these springs, there are some notable differences. For example, we have identified seven clones from the hot spring mats for which the sequence from one end matches closely to some part of the *T. roseum* genome while the sequence from the other end does not have a close match in the *T. roseum* genome. One such clone from Mushroom Spring has a strong match to the *T. roseum* megaplasmid on one end. The other end, which has no good match in the *T. roseum genome*, encodes the CRISPR-associated, RAMP protein, CSM3, which is closely related to CSM3 proteins from other *Chloroflexi*. We believe this is most likely an indication that this clone is from a close relative of *T. roseum* in the environment and that CSM3 has been lost relatively recently from the genome of the strain which we have sequenced. Other interesting findings on clones that are from close relatives of the strain we sequenced include a DNA polymerase that is very distantly related to the DNA polymerase in DSM 5159 as well as a DNA mismatch endonuclease and a DNA methyltransferase not found in DSM 5159. Though it is possible that these chimeric clones are artifacts introduced by the cloning process, the differences are consistent with the sequence diversification and genome-wide rearrangements that are prominent in the hot sprint microbial mats [60].

### Conclusions

As with other genome sequences, analysis of the genome of *T. roseum* reveals many insights into the biology of this organism. For example, genome analysis and experimental studies help refine what we know about this energy metabolism and its novel cell envelope components, as well as other aspects of its biology of this organism. In addition, the genome helps serve as a reference for environmental studies of communities where relatives of this strain are present.

However, this organism was selected for genome sequencing more because of its phylogenetic novelty and not its biology *per se*. Thus, it is important to consider whether such phylogenetically driven genome sequencing is as we expected of value beyond just in aiding studies of a particular organism. On the one hand and in retrospect, this organism was not as phylogenetically novel as we had hoped. This indicates that future phylogenetically driven sequencing projects should probably use the rRNA tree of life directly rather than assignment of organisms to taxonomic groups. Nevertheless, this organism is still distantly related from other organisms for which genomes are available. This distance is important for two reasons. Firstly, it allows the genome to be used to address questions about large-scale evolutionary patterns such as the origin and evolution of the phylum *Chloroflexi* and the evolution of photosynthesis within this group. In addition, and perhaps more importantly, there are some novel features, which are consistent with phylogenetic uniqueness, present in the genome of this organism. For example, this is the first species known to encode all the genes for chemotaxis (including those for motility) on a plasmid. If one's goal is to discover novel features in organisms, certainly sequencing organisms from branches of the tree of life that have not yet been sampled seems to be a good approach. Of course, one cannot always find novelty simply by scanning a genome sequence. However, having a genome sequence from a novel organism is a good way to launch experimental studies of that organism and its relatives which in turn will help identify what novel features they possess.

As of now, there are still hundreds of major branches in the bacterial tree of life that have no genome sequences available for any member. The same is generally true for archaea, eukaryotes, and viruses. We believe that obtaining such genomes is a critical need in order to broaden our understanding of the diversity and evolution of life on this planet.

### Accession Numbers

The annotated genome data reported here is available in Genbank under accession IDs CP001275 (the chromosome) and CP001276 (the megaplasmid).

## Materials and Methods

### Genome sequencing and annotation

Genomic DNA was isolated from exponential-phase cultures of *T. roseum* strain DSM5159. This strain was acquired from the German Collection of Microorganisms and Cell Cultures (DSMZ). Cloning, sequencing, assembly, and closure were performed as previously described [62]. For this sequencing, several shotgun libraries were constructed using inserts with sizes 0.5 kbp, 1.5–3.5 kbp, and 8–14 kbp. In bacteria, the origin of replication is often designated as a region near a cluster of relevant genes (e.g., *dnaN*, *dnaA*, *gyrA*, *gyrB*, *SpooJ*, *gidA*). In *T. roseum*, these genes are dispersed throughout the genome, precluding use of this approach. Therefore, the putative origin was determined from analysis of the GC skew (G−C/G+C) [63]. The transitions between regions of positive and negative GC skew are assumed to correspond to the replication origin and terminus. On that basis, base pair 1 was located in the intergenic region near the DNA polymerase III τ and γ subunits in the circular chromosome, and in the intergenic region near the multiple substrate aminotransferase protein in the megaplasmid. Putative protein coding sequences were identified by Glimmer [64] RNA predictions and the functional annotation of those genes was performed as previously described [62]. The protocols previously built for annotating transport systems using the TransportDB database [36] were adapted and used for transporter analysis.

### Nucleotide composition analysis

Nucleotide composition variations within the *T. roseum* genome were investigated using CompostBin [65], a PCA-based multivariate analysis method. The frequencies of hexamers were extracted from 20,000 random 4 kb fragments sampled from the genome. Each fragment was subsequently represented as a 4096 dimensional vector, for which each component of the vector represents the frequency of one of the 4096 hexamers. Thus, the 20,000 sequences were represented as a 20000*4096 feature matrix. The principal components of the feature matrix were extracted and the projection of the feature matrix in the first two principal components is shown in Figure 2.

### Phylogenetic analysis

For genome tree construction, protein sequences translated from 31 housekeeping genes (*dnaG*, *frr*, *infC*, *nusA*, *pgk*, *pyrG*, *rplA*, *rplB*, *rplC*, *rplD*, *rplE*, *rplF*, *rplK*, *rplL*, *rplM*, *rplN*, *rplP*, *rplS*, *rplT*, *rpmA*, *rpoB*, *rpsB*, *rpsC*, *rpsE*, *rpsI*, *rpsJ*, *rpsK*, *rpsM*, *rpsS*, *smpB*, *tsf*) from genomes of interest were aligned to pre-defined HMM models and ambiguous regions were automatically trimmed according to an embedded mask [62]. A maximum likelihood tree was then built from the concatenated alignments using PHYML [66].

For phylogenetic analysis of individual genes, protein sequences were searched against the NRAA database. Selected sequences were aligned against related Pfam profiles (where applicable); otherwise, alignments were built by MUSCLE [67]. The alignments were trimmed automatically, and then phylogenetic trees were built by PHYML [66]. APIS (Automated Phylogenomic Inference System) was used to build trees for a general phylogenetic survey of the *T. roseum* proteome (Badger et al, in preparation). APIS was implemented as a series of Ruby scripts; the homologs used by APIS for each phylogenetic tree were obtained by comparing each *T. roseum* protein against a curated database of proteins from complete genomes using WU-BLAST [68]. The full-length sequences of these homologs were then retrieved from the database and aligned using MUSCLE [67], and bootstrapped neighbor-joining trees were produced using QuickTree [69]. The inferred trees were then midpoint rooted prior to analysis.

### Metabolic pathway analysis

ECFinder, a system of phylogenetic analysis that is similar to APIS, was employed for genome-wide pathway mapping. ECFinder uses the KEGG database that had been annotated with EC (Enzyme Commission) numbers [70]. Tree-based EC numbers were assigned to the proteins automatically. When members of the query clade did not share the same EC number, the majority EC number was selected. KEGG metabolic pathways were built according to the EC number list obtained for the *T. roseum* genome [70]. Each pathway was examined using the MetaCyc database as references [71]. For pathways omitted from both KEGG and MetaCyc, reference protein sequences were extracted from Genbank, and related Pfam domains were identified [72]. The presence or absence of pathways of interest was determined by BLAST searches of reference sequences and HMM searches of reference Pfam profiles against the *T. roseum* proteome.

### Carotenoid analysis

*T. roseum* was cultured at 65°C and cells were harvested in stationary phase. To produce crude extracts for carotenoid analysis, the pigments were extracted from cell pellets by sonication in 7∶2 acetone∶methanol. Saponification was performed as described previously [73] or, alternatively, cells were suspended in KOH-saturated methanol and incubated overnight at 25°C. The saponified pigments were extracted into ethyl ether, washed, dried under nitrogen, and resuspended in methanol prior to further analysis. Reverse-phase HPLC analysis with compound detection by diode array spectrophotometry was performed as previously described [27].

### CO consumption analysis

*T. roseum* cultures were grown in 120-ml crimp-capped serum bottles with butyl rubber stoppers containing 20 ml of full-strength *T. roseum* medium. The initial headspace was either air or air supplemented with CO to produce an initial CO concentration of about 5% (v/v) or about 20% (v/v). Cultures were grown in the dark on a shaking incubator at 65°C. Samples were collected at different time points for headspace gas analysis and cell counts. Cultures were removed from the incubator for approximately 20 minutes for each sampling. Total protein analysis was performed on the culture samples as a proxy for monitoring the growth stage of the cell cultures. Turbidity reached a plateau around 75 hours, signaling the onset of late exponential phase.

### Metagenomic analysis

We used sequencing reads from the microbial mats from Mushroom Spring and Octopus Spring in the Yellowstone National Park for the metagenomic analysis [60]. The metagenomic sequencing reads were searched against the *T. roseum* genome DNA sequences by BLAT [74], and an identity cutoff of a 92% over 300 bp was applied for the search. Chimeric clones were identified if one end of the read mapped to *T. roseum*, and the other end of the read has no genome match at a 70% identity cutoff over 100 bp.

## Supporting Information

Figure S1 Maximum likelihood tree of blue-copper protein representatives.
(1.05 MB TIF)

Figure S2 Presence of relatives of T. roseum in hot springs in Yellowstone National Park. A. “Density” of T. roseum-like organisms (as reflected in the fraction of the reads per sample) in metagenomic data from Mushroom Spring and Octopus Spring in Yellowstone National Park. B. The distribution of the T. roseum like reads along the chromosome and megaplasmid. Reads from different depths and samples are colored according to the scale bar colors defined in A.
(1.19 MB TIF)
