# Supplementary figures and images for: Complete Genome Sequence of the Aerobic CO-Oxidizing Thermophile Thermomicrobium roseum
Source: PLoS One. 2009 Jan 16;4(1):e4207. doi: 10.1371/journal.pone.0004207 (PMC2615216; doi:10.1371/journal.pone.0004207)

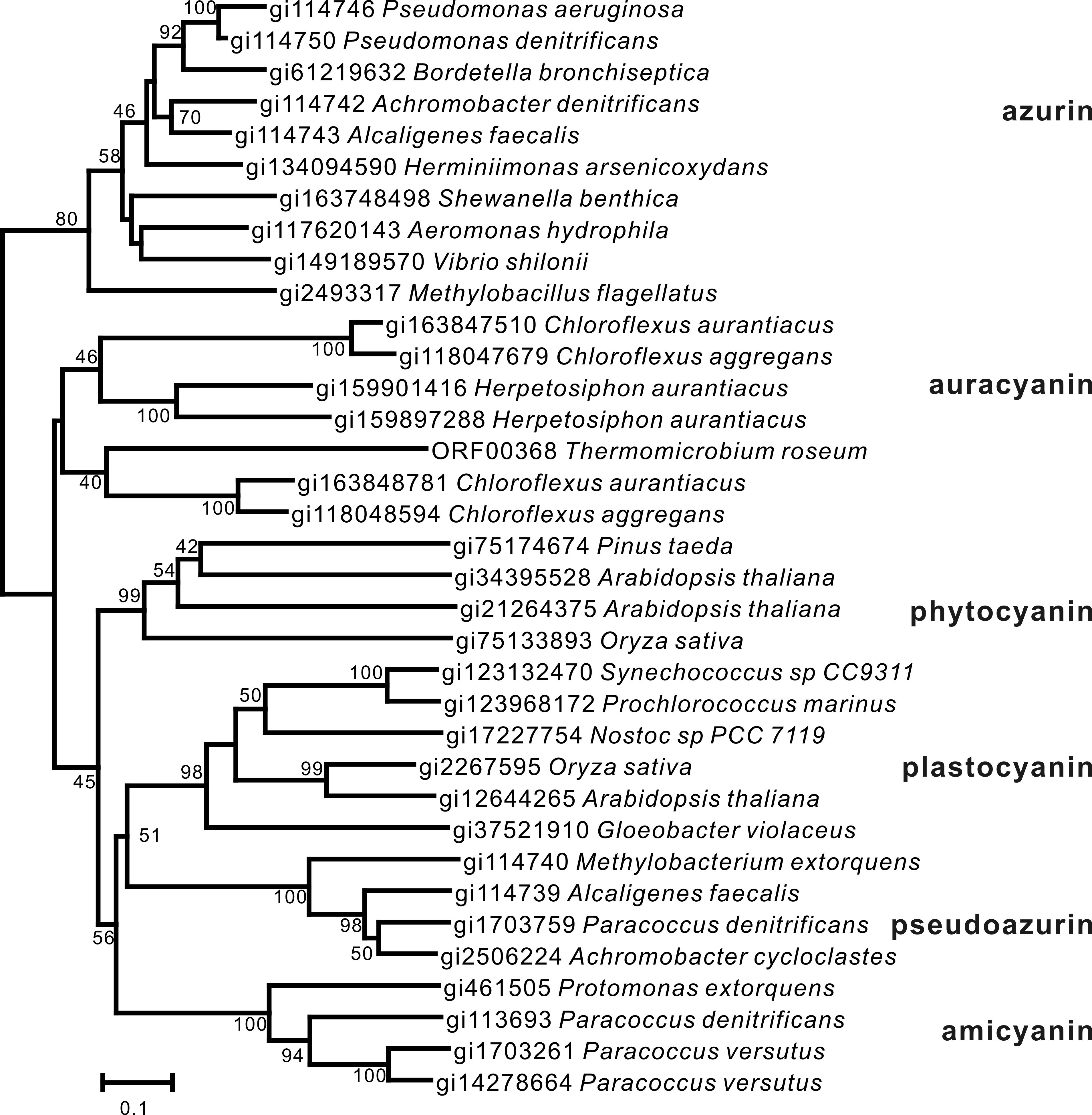

Supplement: Figure S1 — Maximum likelihood tree of blue-copper protein representatives. (1.05 MB TIF) [file pone.0004207.s001.tif]

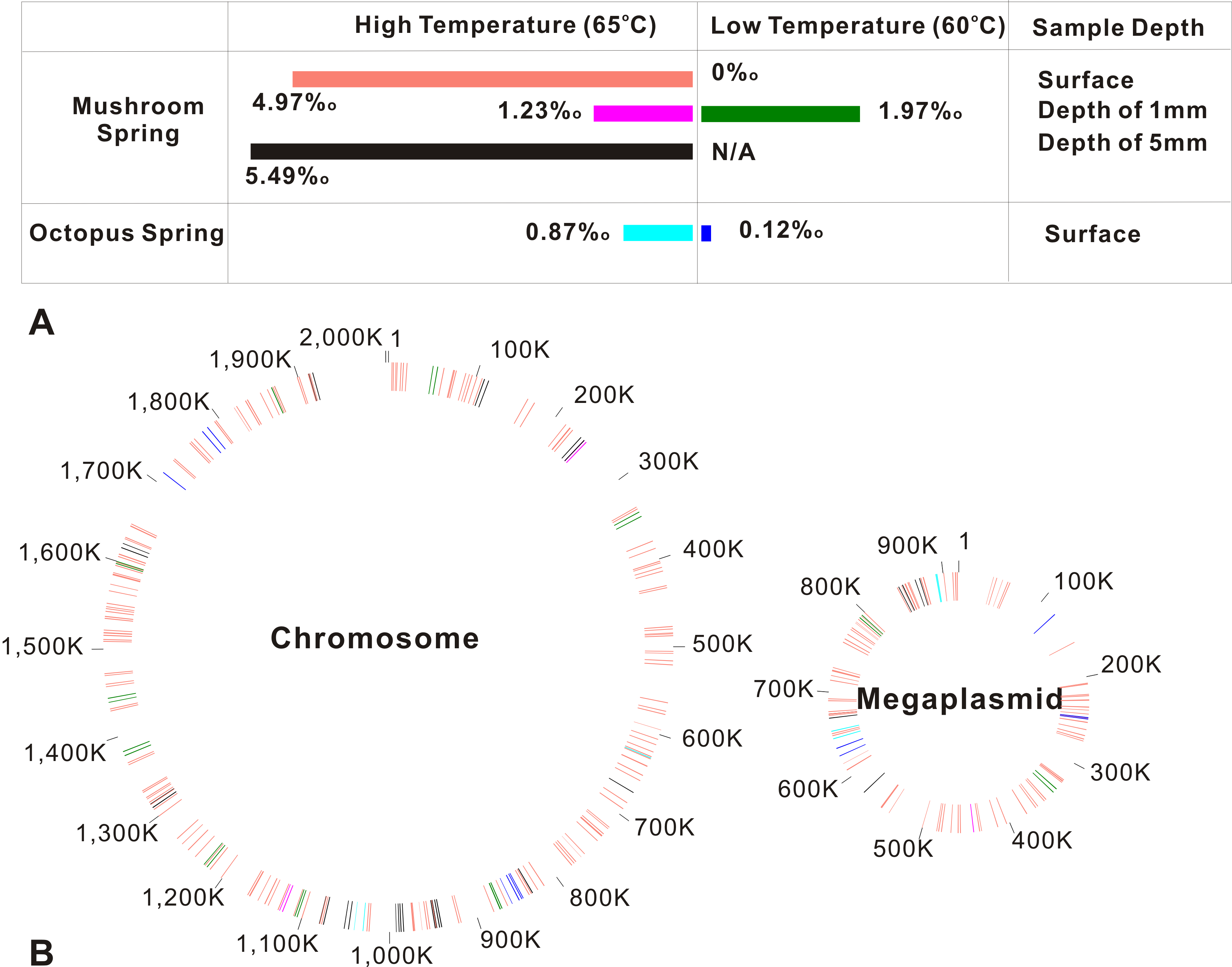

Supplement: Figure S2 — Presence of relatives of T. roseum in hot springs in Yellowstone National Park. A. “Density” of T. roseum-like organisms (as reflected in the fraction of the reads per sample) in metagenomic data from Mushroom Spring and Octopus Spring in Yellowstone National Park. B. The distribution of the T. roseum like reads along the chromosome and megaplasmid. Reads from different depths and samples are colored according to the scale bar colors defined in A. (1.19 MB TIF) [file pone.0004207.s002.tif]
